# Cartilaginous choristoma in the external auditory canal

**DOI:** 10.1016/j.bjorl.2023.101344

**Published:** 2023-10-14

**Authors:** Junhui Jeong, Mi Jang

**Affiliations:** a National Health Insurance Service Ilsan Hospital, Department of Otorhinolaryngology, Goyang, Korea; b National Health Insurance Service Ilsan Hospital, Department of Pathology, Goyang, Korea

## Introduction

Cartilaginous mass in the bony External Auditory Canal (EAC) is uncommon, although about 50 cases have been reported. These masses originally were reported as chondromas.^1^, ^2^, ^3^ However, in 2005, Lee first referred to such an entity as cartilaginous choristoma.^1^, ^2^ A choristoma is a benign tumor-like growth of normal tissue appearing in an abnormal location.^2^, ^3^ Although cartilaginous choristoma in the EAC is rare, otolaryngologists should be aware of this lesion considering its specific histopathologic characteristics. Cartilaginous choristoma in the EAC in a 53-year-old woman is presented here.

## Case report

**Fig. 1:**
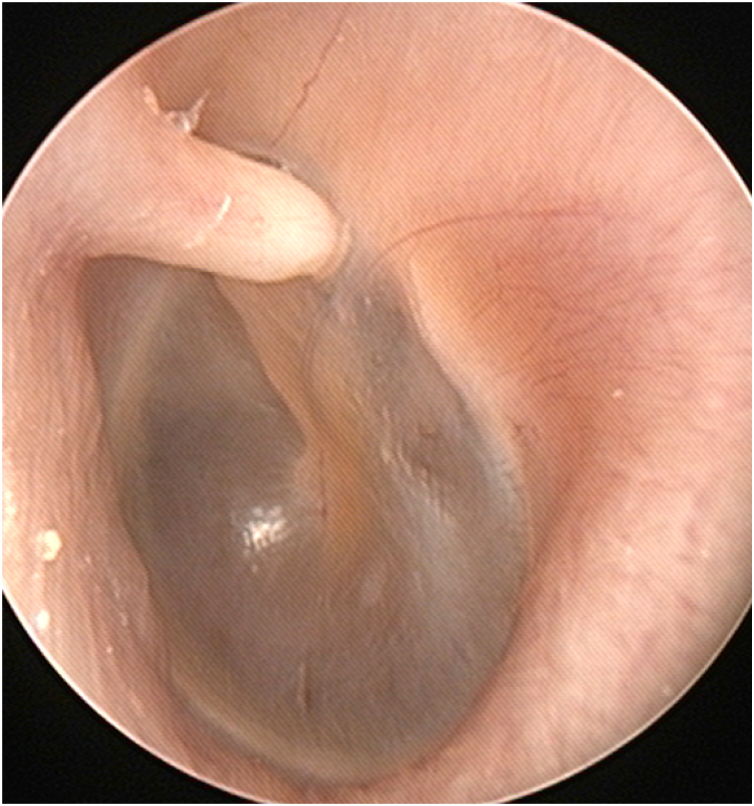
A white, horn-shaped, firm mass in front of the short process of the malleus in the left anterior bony external auditory canal.

**Fig. 2:**
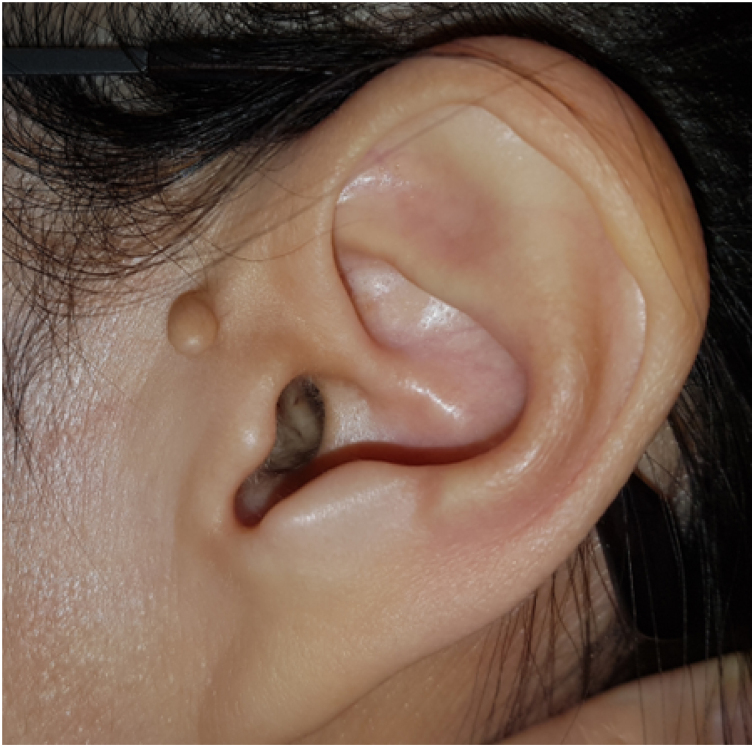
A soft accessory lobe in front of the left tragus.

A 53-year-old woman visited the otorhinolaryngologic clinic for intermittent pain in the left ear for several months. On physical examination, a white, horn-shaped, firm mass was observed in front of the short process of the malleus in the left anterior bony EAC (Fig. 1). A soft accessory lobe was observed in front of the left tragus (Fig. 2). Temporal bone computed tomography showed a 0.3 × 0.1-cm, soft tissue lesion originating from the left anterior bony EAC and no erosion in the cortex of the EAC. Surgical excision was planned because the mass could explain the symptom.

**Fig. 3:**
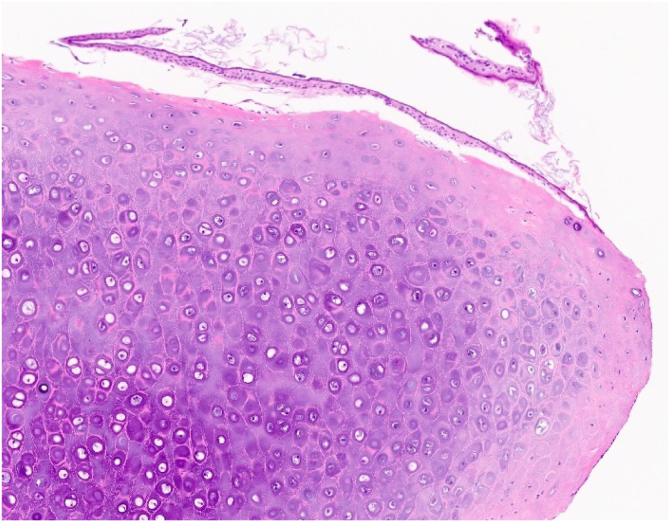
Histopathologic image revealing a mass composed of mature hyaline cartilage covered with squamous epithelium (Hematoxylin-Eosin, ×100).

In surgery under local anesthesia, the 0.3 × 0.1-cm mass in the left anterior bony EAC was removed. The mass was not in contact with the underlying periosteum, and there was no erosion in the periosteum or cortex of the bony EAC. Despite contact with the mass, the eardrum was intact after mass excision. In histopathology, the mass was composed of mature hyaline cartilage covered with squamous epithelium (Fig. 3). Thus, cartilaginous choristoma was diagnosed. There was no recurrence up to six months after surgery.

## Discussion

In 2005, Lee explained why cartilaginous lesions in the bony EAC were cartilaginous choristomas rather than chondromas. First, such masses occurred in tissue not typically containing cartilage. Second, the lesion did not arise from the periosteum but was in contact with the periosteum without erosion. Third, most such lesions were asymptomatic. Lastly, 19 percent of cases had similar congenital external ear malformation of the accessory lobe in front of the tragus, suggesting similar embryological developmental errors in the EAC.^1^ Several types of choristoma have been reported in the head and neck, including gastric mucosa in the tongue, salivary gland tissue in the middle ear, and osseous or cartilaginous mass in the intraoral soft tissue.^2^

A cartilaginous choristoma is commonly small (1–2 mm), solitary, white, smooth, and firm.^3^, ^4^, ^5^ The shape can vary from round to club- or horn-shaped.^3^ Choristoma is typically located in the medial portion of the anterior bony EAC in front of the short process and handle of the malleus.^1^, ^2^, ^4^, ^5^ The mass is typically 1–4 mm in diameter and located 1–3 mm lateral to the eardrum.^1^ Most cases have been diagnosed in the second to fourth decades of life and occurred in Asians.^3^ Most of the lesions were asymptomatic, although repetitive otitis externa is possible.^1^, ^4^

The pathogenesis of cartilaginous choristoma is thought to be migration of embryological cartilage cells.^2^ It is assumed that the mass develops from heterotopic cartilaginous embryonic nests of Meckel’s or Reichert’s cartilage of the first or second branchial arch.^1^, ^2^ The cartilage precursor cells may migrate into the primitive EAC during invagination of the first pharyngeal cleft and grow in the EAC. However, the origin of cartilaginous choristoma remains uncertain.^2^

The treatment of choice is local excision.^3^, ^4^, ^5^ However, cartilaginous choristoma simply can be observed until the lesion causes symptoms.^1^, ^4^ Observation is possible because cartilaginous choristoma has little growth potential, and there have been no reports of malignant transformation.^2^, ^3^ There have been no reports of recurrence after excision.^1^, ^3^

Chondroma can occur from skeletal or extra-skeletal soft tissue. Chondromas from skeletal structures are divided into enchondromas and periosteal chondromas (ecchondromas). Periosteal chondroma is a benign hyaline cartilage neoplasm of the bone surface arising from the periosteum and shows growth potential.^2^ Both cartilaginous choristoma and chondroma are histologically similar to normal and non-neoplastic hyaline cartilage.^3^ Intraoperative findings are essential to distinguish between cartilaginous choristoma and periosteal chondroma; the underlying periosteum and cortex should be eroded in periosteal chondroma, whereas the periosteum and cortex should be normal in cartilaginous choristoma.^2^ Other differential diagnoses of a solitary mass in the EAC include exostosis, osteoma, and cholesteatoma.^3^, ^5^

If a horn-shaped cartilaginous mass is observed in the anterior bony EAC, cartilaginous choristoma should be considered for diagnosis. Although the exact etiology is unclear, this lesion may develop from heterotopic cartilaginous cell nests due to embryological developmental faults. Accompanied external ear malformation of the accessory lobe in front of the ipsilateral tragus aids in diagnosis. The mass can be excised if it produces symptoms.

## Conclusion

A horn-shaped cartilaginous mass in the anterior bony EAC may be a cartilaginous choristoma. Both intraoperative and histopathological findings can help to differentiate such a mass from periosteal chondroma. Treatment for a symptomatic lesion is complete excision.

## Funding

This research did not receive any specific grant from funding agencies in the public, commercial, or not-for-profit sectors.

## Ethical approval

The Institutional Review Board of the National Health Insurance Service Ilsan Hospital exempted the review of this study (NHIMC 2023-01-007).

## Conflicts of interest

The authors declare no conflicts of interest.

## References

[1] Lee F.P. (2005). Cartilaginous choristoma of the bony external auditory canal: a study of 36 cases. Otolaryngol Head Neck Surg.

[2] Yamahara K., Katsura Y., Egawa Y., Lee K., Ikegami S. (2018). Two cases of cartilaginous choristoma-not chondroma of the bony external auditory canal. Case Rep Otolaryngol..

[3] Sethi H.K., Shpigel M., Alnouri G., Zhou J., Sataloff R.T. (2021). Cartilaginous choristoma of the external auditory canal. Ear Nose Throat J..

[4] Lee F.P., Chao P.Z. (2001). Chondroma of the bony external auditory canal. Otolaryngol Head Neck Surg..

[5] Tanigawa T., Inafuku S., Nakayama M. (2008). Five cases of chondroma involving the external auditory canal. Auris Nasus Larynx..

